# A randomized, blinded, controlled USA field study to assess the use of fluralaner topical solution in controlling canine flea infestations

**DOI:** 10.1186/s13071-017-1971-5

**Published:** 2017-01-19

**Authors:** Cheyney Meadows, Frank Guerino, Fangshi Sun

**Affiliations:** Merck Animal Health, Madison, NJ USA

**Keywords:** Bravecto, Fluralaner, Fleas, Fipronil-methoprene, Dogs

## Abstract

**Background:**

Orally administered fluralaner effectively controls fleas and ticks on dogs for 12 weeks. This study evaluates the flea control efficacy achieved with topically applied fluralaner in dogs.

**Methods:**

This investigator-blinded, multi-center randomized, positive controlled study evaluated flea control efficacy in dogs following a single owner-applied treatment of topical fluralaner. A positive control group received three treatments, at 4-week intervals, of a commercial formulation of fipronil/(*S*)-methoprene. All dogs in households randomized to the fluralaner group were dispensed an initial treatment at enrollment and a second treatment at week 12 for an additional 3-week observation of treatment safety. Households with up to five healthy dogs, all at least 12 weeks of age and weighing at least 2 kg (4.4 lb), were randomized in a ratio of 3:1 of fluralaner to positive control. Within households, one primary dog with at least 10 live fleas at enrollment was randomly selected. Flea counts were performed on all primary dogs every 4 weeks through week 12. Efficacy measurement was based on reduction from baseline flea counts. Treatment was considered effective if geometric mean live flea count reductions at weeks 4, 8, and 12 were 90% or greater and significantly different from counts at enrollment. In addition, for each time point the arithmetic mean live flea counts, the efficacy based on arithmetic means, the number and percentage of dogs with at least a 90% reduction in flea count, and the number and percentage of flea free dogs were calculated. Statistical comparisons were also made between treatment groups.

**Results:**

At 12 sites, across 10 states, 121 households (221 dogs) were randomized to receive fluralaner and 44 households (100 dogs) were randomized to receive the positive control. Fluralaner was demonstrated to be significantly effective (all *P* ≤ 0.0001) at 4 weeks (99.8% reduction), 8 weeks (99.9%), and 12 weeks (99.9%). The positive control was significantly different from baseline (all *P* ≤ 0.0001) and showed a reduction of 81.2% at 4 weeks and was effective at 8 weeks (90.3%) and 12 weeks (93.0%). Arithmetic mean flea count reductions for the fluralaner group at 4, 8, and 12 weeks were 99.8, 99.9, and 99.9%, respectively. For the positive control, arithmetic mean flea count reductions were 58.8, 75.3, and 80.8% at 4, 8, and 12 weeks, respectively. No treatment-related serious adverse events were reported in either group.

**Conclusions:**

Owner-applied topical fluralaner treatment was safe in dogs and provided ≥ 99.8% flea control efficacy for 12 weeks.

## Background

The fluralaner chewable tablet (Bravecto® chewable tablet, Merck Animal Health, Madison, NJ) sets a high bar for flea efficacy for a topical follow-on product. In dogs experimentally infested with fleas, the fluralaner chewable tablet showed early onset of flea killing at 2 h and 100% flea killing at 12 h post-administration [1]. This high level of efficacy was sustained for 12 weeks, with 98.0% to 100% reductions in mean live flea counts at 8, 12, and 24 h after experimental reinfestations. A separate laboratory study found that 100% efficacy against fleas was maintained for 122 days post-treatment, with a corresponding elimination of flea egg laying [2]. The prolonged efficacy of oral fluralaner treatment demonstrated in laboratory studies translated into similar benefits under field conditions: a single oral fluralaner treatment of naturally infested dogs provided a significant increase in the percentage of flea-free client-owned dogs compared with three treatments at 28 day intervals with orally administered spinosad [3]. In another study, a single fluralaner treatment resolved flea allergy dermatitis (FAD) signs and reduced owner-assessed scores of their dogs’ pruritus [4]. Fluralaner acaricidal activity was shown in laboratory studies where a single oral administration produced 97.9% efficacy against *Ixodes ricinus* within 8 h of treatment, and 100% at 12 h [5]. Fluralaner is approved in many countries, including the United States and throughout Europe, to provide 12 weeks activity against several tick genera. The rapid onset of fluralaner acaricidal activity was shown to prevent the risk of *Dermacentor reticulatus* transmission of *Babesia canis* in a laboratory study [6]. Pharmacokinetic studies have shown that topically administered fluralaner is distributed systemically and fleas are exposed at the time of feeding [7].

This field study assessed the efficacy of owner-administered topically applied fluralaner at home for flea control compared with dogs in a positive control group that received three treatments with fipronil/(*S*)-methoprene (Frontline® Plus for Dogs, Merial Limited, Duluth, Georgia).

## Methods

The study protocol, finalized in early 2013, used then-current guidelines for evaluating flea and tick parasiticides [8]. It also complied with Good Clinical Practice (VICH GL9) and the International Guiding Principles for Biomedical Research Involving Animals. Written informed consent was obtained from each owner for all household dogs prior to any screening activities. Enrollment eligibility included households that had no more than five dogs, all of which were at least 12 weeks of age, weighed at least 2 kg, and were in generally good health; and that at least one dog in the household had a minimum of 10 live fleas counted prior to enrollment. There were no breed or gender restrictions, but households with pregnant or lactating dogs were not eligible for enrollment. Households in which dogs had exposure to non-confined pets, other than dogs, that could harbor fleas (e.g. cats) were not eligible. There were also restrictions on the pre-enrollment/historical use of any approved (in the USA, as this was a USA study) flea control medications or products, based on the approved label duration. Products labeled for 12-week use had a minimum 84-day washout, products labeled for monthly use had a minimum 30-day washout, products labeled for used every 2 weeks had a minimum 14-day washout, and products labeled for weekly use had a 7-day washout.

Within each of the 12 participating clinics, enrolled households were randomly assigned between two treatment groups. No single clinic was permitted to contribute more than 40% of the households assigned to either group. The groups were:

1. Fluralaner (28% w/v) topical solution for dogs, dispensed for owner administration on Day 0. The efficacy evaluation phase of the study lasted for 12 weeks. A second dose was dispensed after the 12-week efficacy evaluation and dogs were followed for an additional 3 weeks (21 days, until Day 105, Week 15) for additional safety assessment. At least 100 households were targeted for assignment to the fluralaner group. The product was provided to clinics as single-dose applicators in five sizes, containing 112.5 mg, 250 mg, 500 mg, 1,000 mg or 1,400 mg, in volumes of 0.4, 0.89, 1.79, 3.57 and 5 ml, respectively. The targeted dose range for each dog was 25–56 mg/kg.
2. Fipronil/(*S*)-methoprene spot-on solution was dispensed for application once every 28 days for three doses. A minimum of 33 households were targeted for enrollment into this group. This product remained in its commercial packaging containing volumes of 0.67, 1.34, 2.68 or 4.02 ml.

At each site, households were assigned to treatment according to a randomized complete block design, with order of entry into the study as the blocking factor and assignment of dogs to treatment within blocks in a ratio of 3:1 of fluralaner to positive control households. A primary dog from each household was randomly selected from dogs with at least 10 live fleas on initial examination. Separate randomization tables were provided to each site for assignment of households to treatment group and selection of the primary dog. All dogs in a household were assigned to the same treatment group.

Each clinic had at least one dispensing administrator who was responsible for randomization and dispensing all treatments to owners, and ensured that blinded personnel remained blinded by dispensing study products in paper bags. Administrators did not participate in the collection or recording of flea count data or the assessment of FAD. Study personnel who participated in the collection or recording of flea count data or the assessment of FAD through the final visit were masked to treatment assignment.

All treatments were administered at home by dog owners, who were not blinded. For households randomized to the fluralaner group, owners were provided instructions on application using the tube, including parting the hair, placing the tip on the skin and squeezing out the contents onto one or more spots in amounts that would limit risk of topical dispersion from any treatment location. Owners of dogs randomized to the fipronil-methoprene group were instructed to dose according to label directions.

Enrollments were completed during the first clinic visit, during which treatments were dispensed and blood and urine samples collected for baseline clinical pathology data. Owners were required to bring their dogs into the clinic for re-examination visits approximately 4, 8, and 12 weeks after enrollment (on Days 28 [±2], 56 [±3] and 84 [±3]). For households randomized to fipronil-methoprene treatment, study participation ended at 12 weeks. For households randomized to the fluralaner group, dogs were retreated with topical fluralaner at 84 days and were required to return for examination at Day 105 (±3), 3 weeks after the second treatment.

From enrollment until week 12 (Day 84), owners were asked to avoid any premise treatments for environmental flea control, either in their house or on their property. No concomitant treatments for flea and/or tick infestations were permitted during the study period, and the investigator or designee was asked to observe an adequate washout period (consistent with any US-approved labeling) for any such treatments received prior to enrollment. Concomitant treatments for disorders other than flea and tick infestations were permitted, if they were not expected to interfere with assessments of flea and tick infestations. For example, treatments directed at the prevention and control of internal parasite infestations (including heartworm and gastrointestinal parasites) were permitted, if any product used was not labeled for flea or tick control. Treatment that could affect assessment of signs of FAD (for example, steroids, antihistamines, creams, ointments, baths, etc*.*) was permissible. However, data from any such treated dog were excluded from the summaries of signs of FAD after treatment. Grooming, bathing, swimming, and other water activities were permitted during the study, with some exceptions. To avoid any effect on recovery of fleas and ticks, grooming and bathing were not allowed within 72 h before any scheduled re-examination through 12 weeks (Day 84). In addition, bathing, swimming, and other water activities were not allowed for 72 h after application of any study treatment.

Owners were instructed to observe their dogs for any adverse events (AEs), and to document such observations and report them as soon as they occurred or at the next scheduled visit. At each re-examination, primary dogs received a flea count using a flea comb for at least 10 min. The flea count could stop when the counter was confident that all fleas had been recovered.

Skin examinations at each visit included a veterinarian’s assessment of the presence of six signs of FAD (erythema, alopecia, papules, scales, crusts, and excoriations) together with a severity (mild, moderate, severe) assessment.

The primary efficacy endpoint was the reduction in mean flea counts, with household as the experimental unit. Each household was represented by one primary dog.

### Efficacy assessment

Arithmetic and geometric mean live flea counts were calculated separately for each treatment group at each assessment and the percentage reduction at each time point was based on a comparison to baseline according to the formula:

$$ \mathrm{Percent}\;\mathrm{efficacy}=\left(1-\frac{{\mathrm{D}}_{\mathrm{x}}}{{\mathrm{D}}_0}\right)\times 100 $$

where D_0_ = mean live flea count at baseline of primary dogs; and D_x_ = mean live flea count on Day x (x = 28 [4 weeks], 56 [8 weeks] or 84 [12 weeks]) of primary dogs.

Both geometric means and arithmetic means were compared using the live flea counts and log-transformed live flea counts (log [live flea count + 1]) for each household (represented by one primary dog). A mixed linear model with repeated measures was used for the analysis. The model included treatment, visit and treatment*visit as fixed effects, site as a random effect, and household as the subject with repeated measures. A Kenward-Rogers adjustment was used to determine the denominator degrees of freedom for hypothesis testing. Akaike’s Information Criterion (AIC) was used as the criterion to select the covariance structure for repeated measures. Least squares means were used for comparisons and for the log-transformed data, the least squares means were back-transformed to obtain the estimates of geometric mean live flea counts. Within each treatment group, the live flea counts at each post-treatment visit (Day 28, 56 and 84) were compared with that at the baseline (Visit 1). At each visit, live flea counts were compared between the two treatment groups. Two-sided t-tests at a 5% level of significance were used for all pairwise comparisons. SAS version 9.3 was the primary software used for analysis. Treatment was considered effective at a given time point if the mean (geometric or arithmetic) live flea count reduction was 90% or greater and significantly different (*P* ≤ 0.05) from baseline.

Numbers and percentages of primary dogs that showed at least 90% reduction in flea burden, as well as the numbers and percentages of primary dogs with zero fleas counted in each treatment group at each visit were calculated. A non-parametric asymptotic approach was used to test the differences of the percentages between treatment groups. The non-parametric analyses were performed using StatXact version 9. The study was only designed to perform statistical comparisons of the flea counts. Signs of FAD and AEs were only examined descriptively. Thus, no *P*-values were presented for these outcomes.

## Results

Between May and October 2013, 321 dogs from 165 households were enrolled at 12 sites across 10 states: Alabama (one site), Florida (one), Illinois (one), Indiana (one), Kansas (one), Louisiana (one), Maine (one), Pennsylvania (two), Tennessee (one), and Texas (two). There were 121 households (i.e. 121 primary dogs) with a total of 221 dogs randomized to the fluralaner group (51.2% single-dog households), and 44 households with a total of 100 dogs randomized to the fipronil-methoprene group (36.4% single-dog households). Gender distribution, age ranges, and body weights were generally similar between the groups (Table 1). The youngest dogs enrolled in the study were 13 weeks of age in the fluralaner group and 14 weeks of age in the fipronil-methoprene group; 4.5% of fluralaner dogs and 10.0% of fipronil-methoprene dogs were less than 26 weeks. Mixed breeds comprised 35.3 and 40.0% of fluralaner and fipronil-methoprene dogs, respectively.

**Table 1:** Demographics of enrolled dogs and distribution of numbers of dogs in each household

		Fluralaner topical solution (*n* = 221)	Fipronil/(S)-methoprene spot-on solution (*n* = 100)
Age (years)	Mean (SD)	4.5 (3.41)	4.9 (3.70)
	Range	0.3^a^−17.0	0.3^b^−16.3
Weight (lb)	Mean (SD)	35.0 (25.84)	37.8 (29.08)
	Range	4.4−112.0	4.4−136.0
Gender	Female, Intact	41 (18.6%)	21 (21.0%)
	Female, Spayed	70 (31.7%)	29 (29.0%)
	Male, Intact	60 (27.1%)	25 (25.0%)
	Male, Neutered	50 (22.6%)	25 (25.0%)
Distribution of household sizes (no. of dogs)			
	1	62 (51.2%)	16 (36.4%)
	2	30 (24.8%)	14 (31.8%)
	3	19 (15.7%)	5 (11.4%)
	4	8 (6.6%)	4 (9.1%)
	5	2 (1.7%)	5 (11.4%)

^a^in the fluralaner group, the youngest dogs enrolled were 13 weeks of age; 10 (10/221 = 4.5%) were less than 26 weeks of age

^b^in the fipronil group, the youngest dogs enrolled were 14 weeks of age; 10 (10/100 = 10.0%) were less than 26 weeks of age

In both treatment groups, primary dogs occasionally missed visits during this field study, and therefore did not have flea count data generated. In the fluralaner group, there were six dogs that missed at 4 weeks, 12 dogs that missed at 8 weeks, and 11 dogs that missed at 12 weeks. In the fipronil-methoprene group, there were seven dogs that missed at 4 weeks, eight dogs that missed at 8 weeks, and eight dogs that missed at 12 weeks. In addition, in both treatment groups there were some primary dogs for which flea count data were generated, but the results excluded from flea efficacy calculations for reasons including bathing within 72 h prior to a flea count or 72 h after administration of a treatment, incorrect dosing, or insecticidal treatment of the household. In the fluralaner group, data from one primary dog were excluded at the initial count, data from five dogs were excluded at 4 weeks, data from four dogs were excluded at 8 weeks, and data from six dogs were excluded at 12 weeks. In the fipronil-methoprene group, data from one primary dog were excluded at 4 weeks and data from three dogs were excluded at 12 weeks.

**Fig. 1:**
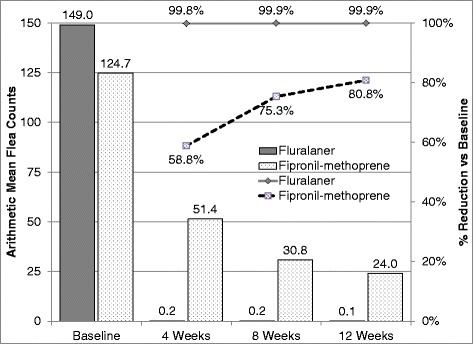
Arithmetic mean flea counts and percent reduction from baseline to weeks 4, 8 and 12 for topical fluralaner or fipronil/(*S*)-methoprene treated dogs (bars indicate arithmetic flea counts; lines indicate percentage reductions from baseline)

In the fluralaner group, treatment was demonstrated to be effective by geometric means (percent reduction ≥ 90% *vs* baseline) at 4 weeks (99.8% reduction), 8 weeks (99.9%) and 12 weeks (99.9%), and flea count reductions were significantly different from baseline (all *P* < 0.0001) (Table 2). Arithmetic mean efficacy of the fluralaner group was 99.8%, 99.9% and 99.9% at weeks 4, 8, and 12, respectively (Table 2, Fig. 1, all *P* < 0.0001). The percentages of individual fluralaner-treated primary dogs with a ≥ 90% reduction in flea burden from baseline were 100% at 4 weeks, 100% at 8 weeks, and 98.1% at 12 weeks. The percentages of individual fluralaner-treated primary dogs with 0 fleas detected (i.e. a 100% reduction) were 84.5% at 4 weeks, 93.3% at 8 weeks, and 93.3% at 12 weeks (Table 2).

**Table 2:** Flea counts for primary dogs enrolled in a field flea-efficacy evaluation of fluralaner topical solution and fipronil/(S)-methoprene. No efficacy comparison was performed at V1 and no primary dogs had 90% reduction or were flea-free at V1

	Visit 1 (enrollment)	Visit 2 (Week 4, Day 28)	Visit 3 (Week 8, Day 56)	Visit 4 (Week 12, Day 84)
Number of primary dogs				
Fluralaner topical solution	120	110	105	104
Fipronil/(S)-methoprene spot-on solution	44	36	36	33
Arithmetic mean flea count (95% CI)				
Fluralaner topical solution	149.0 (109.9–188.2)	0.2 (0.1–0.4)	0.2 (0.0–0.3)	0.1 (0.0–0.2)
Fipronil/(S)-methoprene spot-on solution	124.7 (78.1–171.3)	51.4 (16.9–86.0)	30.8 (10.8–50.9)	24.0 (3.6–44.4)
*P-*value for comparison^a^	*t*_(161.8)_ = 0.67, *P* = 0.5011	*t*_(152.3)_ = -4.96, *P* < 0.0001	*t*_(150.3)_ = –5.35, *P* < 0.0001	*t*_(106.9)_ = -4.22, *P* < 0.0001
% efficacy (reduction from baseline) based on arithmetic means				
Fluralaner topical solution	na	99.8	99.9	99.9
*P-*value for comparison to baseline^a^	na	*t*_(162.9)_ = 8.04, *P* < 0.0001	*t*_(162.0)_ = 8.05, *P* < 0.0001	*t*_(161.5)_ = 7.95, *P* < 0.0001
Fipronil/(S)-methoprene spot-on solution	na	58.8	75.3	80.8
*P-*value for comparison to baseline^a^	na	*t*_(164.4)_ = 2.51, *P* = 0.0132	*t*_(162.4)_ = 3.10, *P* = 0.0023	*t*_(162.6)_ = 2.93, *P* = 0.0039
Geometric mean flea count (95% CI)				
Fluralaner topical solution	68.9 (55.2–85.9)	0.1 (0.1–0.2)	0.1 (0.0–0.1)	0.1 (0.0–0.1)
Fipronil/(S)–methoprene spot-on solution	66.6 (47.6–93.1)	12.5 (6.7–22.8)	6.4 (3.1–12.4)	4.7 (2.2–9.0)
*P-*value for comparison^a^	*t*_(158.2)_ = 0.154, *P* = 0.8782	*t*_(145.6)_ = -14.13, *P* < 0.0001	*t*_(145.9)_ = -10.77, *P* < 0.0001	*t*_(134.4)_ = -10.17, *P* < 0.0001
% efficacy (reduction from baseline) based on geometric means				
Fluralaner topical solution	na	99.8	99.9	99.9
*P-*value for comparison to baseline^a^	na	*t*_(158.1)_ = 34.41, *P* < 0.0001	*t*_(156.3)_ = 32.47, *P* < 0.0001	*t*_(155.7)_ = 31.83, *P* < 0.0001
Fipronil/(S)-methoprene spot-on solution	na	81.2	90.3	93.0
*P-*value for comparison to baseline^a^	na	*t*_(165.5)_ = 8.32, *P* < 0.0001	*t*_(161.1)_ = 10.40, *P* < 0.0001	*t*_(165.5)_ = 10.95, *P* < 0.0001
% of primary dogs with at least 90% reduction from baseline flea count				
Fluralaner topical solution	na	100.0	100.0	98.1
Fipronil/(S)-methoprene spot-on solution	na	38.9	55.6	54.5
*P-*value for comparison^b^	na	*Z* = -9.11, *P* < 0.0001	*Z* = -7.26, *P* < 0.0001	*Z* = -6.61, *P* < 0.0001
% of flea-free primary dogs				
Fluralaner topical solution	na	84.5	93.3	93.3
Fipronil/(S)-methoprene spot-on solution	na	13.9	30.6	30.3
*P-*value for comparison^a^	na	*Z* = -7.83, *P* < 0.0001	*Z* = -7.76, *P* < 0.0001	*Z* = -7.62, *P* < 0.0001

^a^ *P*-value for comparison of model least squares means parameter estimates

^b^ *P*-value for comparison of percentages using non-parametric asymptotic approach and Standardized Statistic

*Abbreviation*: *na* value or calculation is not applicable

In the fipronil-methoprene group, treatment was demonstrated effective based on geometric means at weeks 8 (90.3%) and 12 (93.0%) (Table 2), and were significantly different from baseline (all *P* < 0.0001). At 4 weeks, the reduction by geometric means was 81.2%, which was less than 90% but was significantly different from baseline (*P* < 0.0001). Arithmetic mean efficacy of the fipronil-methoprene group was 58.8, 75.3 and 80.8% at weeks 4, 8 and 12, respectively (Table 2, Fig. 1, all *P* ≤ 0.0132). The percentages of individual fipronil-methoprene-treated primary dogs with a ≥ 90% reduction in flea burden from baseline were 38.9% at 4 weeks, 55.6% at 8 weeks, and 54.5% at 12 weeks. The percentages of individual fipronil-methoprene-treated primary dogs with 0 fleas detected (i.e. a 100% reduction) were 13.9% at 4 weeks, 30.6% at 8 weeks, and 30.3% at 12 weeks.

In both groups, there was improvement of FAD signs in both the fluralaner topical and positive control treated groups (Table 3). The most common manifestation of FAD observed in study dogs was erythema, which at enrollment was recorded for 83 of 221 (37.6%) dogs in the fluralaner group and 36 of 100 (36.0%) dogs in the fipronil-methoprene group. At 12 weeks, the percentage of eligible dogs in which this sign was seen to have resolved was 91.4% in the fluralaner and 60.0% in the fipronil-methoprene group.

**Table 3:** Proportion of dogs showing Flea Allergy Dermatitis signs at the initial visit and 12 week recheck following treatment with either topical fluralaner or a positive control

Sign		Fluralaner topical solution	Fipronil/(*S*)-methoprene spot-on solution
Erythema	Number of dogs with sign at initial exam that were also eligible for re-examination at 12 weeks	58	25
	% of dogs with lesion resolved at 12-week re-examination	91.4	60.0
Alopecia	Number of dogs with sign at initial exam that were also eligible for re-examination at 12 weeks	43	19
	% of dogs with lesion resolved at 12-week re-examination	86.0	78.9
Papules	Number of dogs with sign at initial exam that were also eligible for re–examination at 12 weeks	32	6
	% of eligible dogs with lesion resolved at 12-week re-examination	96.9	100.0
Scales	Number of dogs with sign at initial exam that were also eligible for re-examination at 12 weeks	27	11
	% of eligible dogs with lesion resolved at 12-week re-examination	92.6	72.7
Crusts	Number of dogs with sign at initial exam that were also eligible for re-examination at 12 weeks	28	10
	% of eligible dogs with lesion resolved at 12-week re-examination	100.0	80.0
Excoriation	Number of dogs with sign at initial exam that were also eligible for re-examination at 12 weeks	25	6
	% of eligible dogs with lesion resolved at 12-week re-examination	100.0	83.3

No serious adverse events were reported in either study group. All adverse events were unremarkable throughout the study. A review of study records and owner diaries showed that vomiting was the most frequent event in both groups, affecting 6.3% of fluralaner treated dogs and 6.0% of fipronil-methoprene treated dogs. Other adverse events occurred at generally similar rates in both groups, although diarrhea was reported in 11.0% of fipronil-methoprene treated dogs and 2.7% of fluralaner treated dogs; alopecia (focal hair loss) and decreased appetite were reported in more fluralaner treated dogs (4.1 and 2.0%, respectively) than fipronil-methoprene treated dogs (1.4 and 0.0%, respectively) (Table 4).

**Table 4:** Percent of dogs experiencing most common adverse events reported during the study

	Fluralaner topical solution (*n* = 221)	Fipronil/(*S*)-methoprene spot-on solution (*n* = 100)
Vomiting	6.3	6.0
Alopecia	4.1	2.0
Diarrhea	2.7	11.0
Lethargy	2.7	2.0
Decreased appetite	1.4	0.0
Moist dermatitis/Rash	0.9	0.0

Clinical pathology for both groups was unremarkable, with no clinically relevant observations suggestive of pathologic trends, and only occasional isolated departures from normal reference ranges in blood and urine analyses. There were no clinically relevant clinical pathology differences between the fluralaner and fipronil-methoprene groups.

## Discussion

Treatment with topical fluralaner provided a high and persistent level of flea control efficacy throughout the 12-week duration of efficacy evaluation. This efficacy was greater than the flea control efficacy of the fipronil-methoprene treatment at several time points. Results of this field study seen with the topical fluralaner formulation align with results of a study with a similar design that assessed the chewable fluralaner formulation [3]. In both studies, a single treatment/application resulted in a > 99% reduction in live flea counts within 4 weeks, and sustained flea count reductions of > 99% through 12 weeks; more than 80% of fluralaner-treated dogs were free of fleas at 4 weeks, and more than 90% were free of fleas at the 8- and 12-week assessments.

The overall flea control results for fluralaner compared favorably with those of fipronil-methoprene. Furthermore, the results observed are also consistent with findings in other field studies. For instance, in the aforementioned field trial with the oral fluralaner formulation, the oral fluralaner group had > 90% of dogs flea-free after a single treatment [3]. In an earlier spinosad study in which fipronil-methoprene was a comparator product, only 38% of dogs in the fipronil-methoprene group were free of fleas after three consecutive monthly treatments [9], aligning with our results of 30% of flea-free dogs receiving three owner-applied fipronil-methoprene treatments at 4 week intervals. Another 2013 study comparing indoxacarb to fipronil-methoprene for flea control in dogs and cats in private homes showed that 16% of fipronil-methoprene-treated pets were flea free after two owner-applied monthly treatments [10].

There were households in this study that failed to achieve adequate flea control in fipronil-methoprene treated dogs. Previous reports [9, 10] suggested that treatment failures with fipronil /(*S*)-methoprene may have been caused by: inherent variability of topical administration linked to incomplete owner compliance with dosing directions; to effects of bathing; to climatic conditions; or to other unknown factors. In this current study, the fact that topically-applied fluralaner was highly effective suggest that owners in the study understood topical application and reduce the likelihood that owner misapplication explains the results observed in the fipronil-methoprene dogs. Also, bathing or exposure to water within specified time periods relative to dosing was documented in this study, and data points affected by these events were appropriately excluded. Furthermore, household flea control failures for fipronil-methoprene dogs occurred in both southern enrolling clinics (in a warmer climate that would be expected to have substantial flea burdens), and in northern clinics, where generally lighter flea burdens might be expected.

As would be expected from the use of highly effective flea control measures, and matching findings in earlier field studies, treatment with topical fluralaner was followed by resolution of FAD signs in treated dogs [3, 4, 11].

Both the topical fluralaner and topical fipronil-methoprene products were well tolerated in this study. There were no detectable effects of either product on clinical pathology tests (baseline and week 12 for fipronil-methoprene dogs; baseline, week 12, and week 15 for fluralaner dogs).

## Conclusions

In conclusion, the fluralaner topical solution was well tolerated in this study and was highly effective in removing flea infestations present at the time of initial treatment, and in preventing reinfestation. The results show that owner applied fluralaner topical solution can provide convenient and reliable flea control for veterinarians and dog owners who prefer the topical route.

## Abbreviation

FAD: Flea allergy dermatitis

## References

[1] Taenzler J, Wengenmayer C, Williams H, Fourie J, Zschiesche E, Roepke RK (2014). Onset of activity of fluralaner (BRAVECTO™) against *Ctenocephalides felis* on dogs. Parasit Vectors..

[2] Dryden MW, Smith V, Bennett T, Math L, Kallman J, Heaney K (2015). Efficacy of fluralaner flavored chews (Bravecto) administered to dogs against the adult cat flea, *Ctenocephalides felis felis* and egg production. Parasit Vectors.

[3] Meadows C, Guerino F, Sun F (2014). A randomized, blinded, controlled USA field study to assess the use of fluralaner tablets in controlling canine flea infestations. Parasit Vectors..

[4] Fisara P, Shipstone M, von Berky A, von Berky J (2015). A small-scale open-label study of the treatment of canine flea allergy dermatitis with fluralaner. Vet Dermatol..

[5] Wengenmayer C, Williams H, Zschiesche E, Moritz A, Langenstein J, Roepke RK (2014). The speed of kill of fluralaner (Bravecto™) against *Ixodes ricinus* ticks on dogs. Parasit Vectors..

[6] Taenzler J, Liebenberg J, Roepke RK, Heckeroth AR (2015). Prevention of transmission of *Babesia canis* by *Dermacentor reticulatus* ticks to dogs treated orally with fluralaner chewable tablets (Bravecto™). Parasit Vectors..

[7] Klip S, Ramirez D, Allan MJ, Roepke RK (2016). Comparative pharmacokinetics of fluralaner in dogs and cats following single topical or intravenous administration. Parasit Vectors..

[8] Marchiondo AA, Holdsworth PA, Green P, Blagburn BL, Jacobs DE (2007). World Association for the Advancement of Veterinary Parasitology (W.A.A.V.P) guidelines for evaluating the efficacy of parasiticides for the treatment, prevention, and control of flea and tick infestation on dogs and cats. Vet Parasitol.

[9] Dryden MW, Ryan WG, Bell M, Rumschlag AJ, Young LM, Snyder DE (2013). Assessment of owner-administered monthly treatments with oral spinosad or topical spot-on fipronil/(S)-methoprene in controlling fleas and associated pruritus in dogs. Vet Parasitol..

[10] Dryden MW, Payne PA, Smith V, Chwala M, Jones E, Davenport J (2013). Evaluation of indoxacarb and fipronil (s)-methoprene topical spot-on formulations to control flea populations in naturally infested dogs and cats in private residences in Tampa FL. USA. Parasit Vectors..

[11] Rohdich N, Roepke RK, Zschiesche E (2014). A randomized, blinded, controlled and multi-centered field study comparing the efficacy and safety of Bravecto (fluralaner) against Frontline (fipronil) in flea- and tick-infested dogs. Parasit Vectors..

